# The number of cycles of adjuvant chemotherapy in stage III and high-risk stage II rectal cancer: a nomogram and recursive partitioning analysis

**DOI:** 10.1186/s12957-022-02582-6

**Published:** 2022-04-12

**Authors:** Wei-Wei Chen, Wen-Ling Wang, Hong-Min Dong, Gang Wang, Xiao-Kai Li, Guo-Dong Li, Wang-Hua Chen, Juan Chen, Sai-Xi Bai

**Affiliations:** 1 Department of Oncology, Affiliated Hospital of Guizhou Medical University, Guiyang, 550000 China; 2 Department of Clinical Medicine, Guizhou Medical University, Guiyang, 550000 China; 3 Department of Abdominal Oncology, Affiliated Cancer Hospital of Guizhou Medical University, Guiyang, 550000 China

**Keywords:** Rectal neoplasm, Chemotherapy, Adjuvant, Prognosis, Nomogram, Recursive partitioning analysis

## Abstract

**Objective:**

The prognostic role of the number of cycles of adjuvant chemotherapy (ACT) after total mesorectal excision in stage III and high-risk stage II rectal cancer is unknown. As a result of this, our study was designed to assess the effect of the number of cycles of ACT on the prediction of cancer-specific survival.

**Methods:**

Four hundred patients that were diagnosed as stage III and high-risk stage II rectal cancer from January 2012 to January 2018 and who had received total mesorectal excision were enrolled in this study. A nomogram incorporating the number of cycles of ACT was also developed in this study. For internal validation, the bootstrap method was used and the consistency index was used to evaluate the accuracy of the model. The patients were stratified into risk groups according to their tumor characteristics by recursive partitioning analysis.

**Results:**

We found that the risk of death was decreased by 26% (HR = 0.74, 95% CI: 0.61–0.89, *P* = 0.0016) with each increasing ACT cycle. The N stage, positive lymph node ratio (PLNR), carcinoembryonic antigen, neutrophil-to-lymphocyte ratio, and the number of cycles of ACT were chosen and entered into the nomogram model. Recursive partitioning analysis-based risk stratification revealed a significant difference in the prognosis in rectal cancer patients with high-risk, intermediate-risk, and low-risk (3-year cancer-specific survival: 0.246 vs. 0.795 vs. 0.968, *P* < 0.0001). Seven or more cycles of ACT yielded better survival in patients with PLNR ≥ 0.28 but not in patients with PLNR < 0.28.

**Conclusion:**

In conclusion, the nomogram prognosis model based on the number of cycles of ACT predicted individual prognosis in rectal cancer patients who had undergone total mesorectal excision. These findings further showed that in patients with PLNR ≥ 0.28, no fewer than 7 cycles of ACT are needed to significantly reduce the patient’s risk of death.

## Introduction

Rectal cancer is a common malignant tumor that seriously threatens the health of Chinese individuals. More than 70% of rectal cancers are initially diagnosed as stage II and stage III diseases [1]. For resectable rectal cancer, total mesorectal excision (TME) is the standard recommended treatment [1] and radiotherapy after surgery has not been proven to benefit the overall survival of patients with rectal cancer [2]. Therefore, there is reason to believe that the main cause of treatment failure is distant metastasis rather than local recurrence. Six months of adjuvant chemotherapy (ACT) is the main approach employed by clinicians to decrease the risk of distant metastases and improve overall survival in patients with colon cancer [3]. However, no consensus has been reached regarding the adjuvant treatment of surgically resectable rectal cancer. The recommended ACT regimens for patients with stage III and high-risk stage II rectal cancer, which consist of oxaliplatin and fluorouracil, are based on research conducted on colon cancer [3]. However, even for colon cancer, studies on the relationship between the number of cycles of ACT and the prognosis of the disease are few [4, 5]. Rectal cancer is also different from colon cancer in clinical behavior and biology [6–8]. As a result, there is insufficient evidence to recommend the optimal number of cycles of ACT in patients with rectal cancer.

Neoadjuvant chemoradiation (NACR) is recommended in rectal cancer, but controversial focus on its role in overall survival. Intensive chemotherapy, which eliminates circulating tumor cells, may decrease the risk of distant metastasis and benefit the survival [9]. However, oxaliplatin is associated with cumulative neurotoxicity. The long duration of chemotherapy might cause severe toxicities and even treatment-related death. Therefore, the appropriate chemotherapy cycle for patients with rectal cancer should be determined.

In our study, we retrospectively analyzed data from patients with rectal cancer who had stage III and high-risk stage II diseases. Multivariate regression analysis adjusted for covariates was used to study the independent effects of the number of cycles of ACT on prognosis. A nomogram model was used to predict cancer-specific survival (CSS). A risk stratification system based on recursive partitioning analysis (RPA) was also adopted to determine the appropriate number of cycles of ACT for various risk groups.

## Materials and methods

### Patient selection and follow-ups

Patients diagnosed with stage III and high-risk stage II rectal cancer from January 2012 to January 2018 were enrolled in this study. All tumors were re-staged according to the eighth edition of the Union for International Cancer Control/American Joint Committee on Cancer tumor-node-metastasis TNM staging [10]. Inclusion criteria: (1) Patients that had pathologically confirmed high-risk stage II (defined as MSI negative disease; tumor obstruction with or without tumor perforation preoperatively; poor differentiation; positive margins; fewer than 12 lymph nodes detected; perineural infiltration; invasion of extramural venous or lymphatic vascular; or T4 disease) or stage III adenocarcinoma; and (2) Patients that had the TME surgery. In total, 400 patients met the inclusion criteria.

Follow-ups were conducted through outpatient visits, including clinical examinations, routine hematologic and chemical blood tests (obtained before surgery), carcinoma antigen 199 (CA199), carcinoembryonic antigen (CEA), colonoscopy, and abdominal and chest computed tomography examinations. Follow-up evaluations were conducted every 3 months for the first 2 years, every half year for the next 3 years, and yearly thereafter.

### Variables

CSS was defined as the duration from the operation to the patient’s death due to rectal cancer or until the last follow-up. The number of cycles of ACT was a continuous variable. The covariates included in this study were (1) demographic data; and (2) factors from prior studies associated with survival [11–14].

The following variables were implemented to construct a multivariable-adjusted model: (1) Continuous variables: age, CA199 (obtained before surgery), CEA (obtained before surgery), positive lymph node ratio (PLNR), prognostic nutritional index (PNI: serum albumin + 5 × lymphocyte count, obtained before surgery), ratio of neutrophils to lymphocytes (NLR, obtained before surgery), and the cycles of ACT; and (2) Categorical variables: gender, T stage, N stage, differentiation, and chemoradiotherapy given or not given.

### The treatment strategy

Concurrent chemoradiation (CCRT) was recommended to all patients, and neoadjuvant treatment was planned to be superior to adjuvant chemoradiotherapy. External beam radiotherapy was administered through 3-dimensional conformal radiotherapy or intensity-modulated radiotherapy. The target volumes were contoured according to the International Commission on Radiation Units and Measurements 50 and 62 guidelines. The radiotherapy range for patients who received NACR included primary tumor, and the local lymphatic drainage area; for patients who had postoperative chemoradiation, the range included tumor bed of the primary tumor, anastomosis, and the local lymphatic drainage area. The total radiation dose was 5000cGy (1 fraction per day, 200 cGy per fraction). Patients with adjuvant chemoradiotherapy received capecitabine (850mg/m^2^, twice daily) or 5-fluorouracil (225 mg/m^2^, continuous intravenous infusion) as the concurrent chemotherapy. The patients enrolled in this study were also recommended to receive a six-month ACT regimen that consisted of oxaliplatin, 5-fluorouracil/leucovorin (FOLFOX, oxaliplatin 85 mg/m^2^, and dl-LV 400 mg/m^2^, followed by bolus 5-FU 400 mg/m^2^ and a 46-48h infusion of 5-FU 2400 mg/m^2^, 1 cycle every 14 days).

### Statistical analysis

We expressed continuous variables as mean ± standard and median (min, max) and expressed categorical variables as frequency (percentage). The relationship between the covariates and outcomes was studied with a univariate cox proportional hazard model. We also used three models to assess the effect of the number of cycles of ACT on CSS: model 1, none of the covariates were adjusted; model 2 (Adjust I), only adjusted for age, gender, and tumor stage; and model 3 (Adjust II), model 2 plus other covariates, as listed in Table 1. For missing records of the covariates, we used dummy variables to indicate missing covariate values. We assigned missing records for each covariate to 0 and additionally created a dummy variable (0 = without missing; 1 = missing). Both variables were entered into the model at the same time (*Y* = *a* X 1 + *b* * dummy variable).

**Table 1:** Clinicopathological characteristics of patients with high-risk stage II and stage III rectal cancer after TME surgery

	Mean (SD), median (min–max)
Age	54.6 (12.8), 56.0 (18.0–85.0)
Gender (*N*, %)	
Male	244 (61.0%)
Female	156 (39.0%)
T stage (*N*, %)	
T1–2	21 (5.25%)
T3–4	379 (94.75%)
N stage (*N*, %)	
N0	182 (45.5%)
N1	140 (35.0%)
N2	78 (19.5%)
PLNR	0.145 (0.23), 0.02 (0.00–1.00)
< 0.145	286 (71.50%)
≥ 0.145	114 (28.50%)
Differentiation (*N*, %)	
Moderate to high	350 (87.5%)
Poor	50 (12.5%)
CA199	26.97 (69.9), 11.1 (0.00–700)
Normal	352 (89.34%)
Elevated	42 (10.66%)
CEA	6.16 (14.5), 1.82 (0.00–101.5)
Normal	312 (78.00%)
Elevated	88 (22.00%)
PNI	49.1 (5.51), 49.00 (27.0–68.2)
PNI tertile 1	132 (33.00%)
PNI tertile 2	134 (33.50%)
PNI tertile 3	134 (33.50%)
NLR	3.21 (3.19), 2.29 (0.72–33.0)
NLR tertile 1	133 (33.25%)
NLR tertile 2	133 (33.25%)
NLR tertile 3	134 (33.50%)
Number of ACT cycle	5.43 (3.17), 6.00 (0.00–12.0)
0	36 (9.00%)
1–6	228 (57.00%)
7–12	136 (34.00%)
CCRT (*N*, %)	
Without	147 (36.8%)
With	253 (63.3%)

Abbreviations: *PLNR* Positive lymph nodes ratio, *CA* Carcinoma antigen, *CEA* Carcinoembryonic antigen, *CCRT* Concurrent chemoradiotherapy, *ACT* Adjuvant chemotherapy, *PNI* Prognostic nutritional index, *NLR* Neutrophil-lymphocyte ratio

A nomogram predicting 3-year CSS was developed using the backward stepwise method. The stepwise process was evaluated with the Bayesian information criterion and the Akaike information criterion. The model with the lowest of these criteria was chosen as the final model. Covariates, such as the N stage, PLNR, CEA, PNI, and the number of cycles of ACT were included in the final model. The predicted performance was quantified by the consistency index, the predictive accuracy was measured by the bootstrap (500 resample) method, and the calibration curve was drawn according to the actual proportions and predicted probabilities.

RPA identified factors to stratify patients into the risk groups based on 3-year CSS; it also found the cutoffs that maximized the differentiation in risk-specific survival [15]. The covariates were further analyzed using the decision tree models generated by RPA. According to the diagnostic sensitivity, specificity, and accuracy, the best decision tree was selected. Kaplan-Meier curves were drawn in each risk group generated by RPA and 3-year CSS between the groups that were stratified by RPA were compared by log-rank tests. We reanalyzed CSS in the RPA-generated risk groups according to the cycles of ACT. All statistical analyses were performed using the R programming language and environment (version 3.6.3, R Foundation for Statistical Computing, Austria) [16]. *P* < 0.05 was considered significant.

## Results

### The baseline clinicopathological characteristics of the patients enrolled in this study

**Fig. 1:**
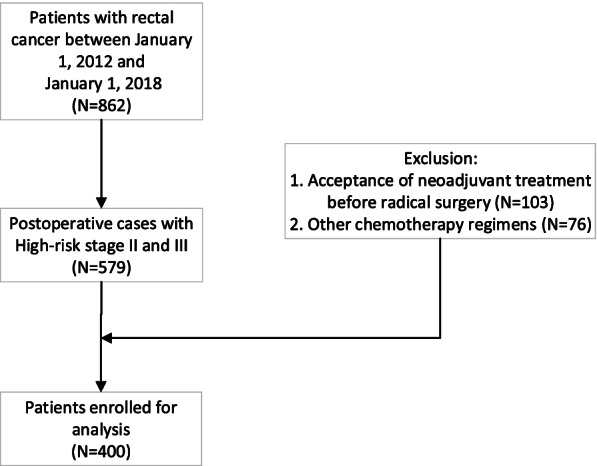
Flow chart of enrollment

The flow chart of the enrolled patients is shown in Fig. 1. The median age of the included patients was 56 years, and 61% were male. 94.75% of the enrolled patients were in stage T3–4, and lymph node metastasis occurred in 54.5% of enrolled patients. The median adjuvant chemotherapy cycle was 6 cycles. The clinicopathological characteristics of the included rectal cancer patients are summarized in Table 1. The median follow-up time was 37.5 months (7.1–90.3 months). By the end of the follow-up period, 11 patients were lost and 34 deaths from rectal cancer were confirmed. The 3-year CSS was 93.25%. At 3 years, the local recurrence and distant metastasis rates were 4.75% and 13.3%, respectively. In total, 253 patients (253/400) received CCRT, and 239 (239/253) had NACR.

### The univariate and multivariate Cox proportional hazard model

Table 2 shows that the N stage, PLNR, PNI, NLR, and the number of cycles of ACT were associated with 3-year CSS in the univariate analysis (*P* < 0.05). Three models were conducted to analyze the independent association of the number of cycles of ACT on postoperative 3-year CSS (multivariate Cox proportional hazard model) (Table 3). In the non-adjusted model (model 1), the model-based hazard ratio (HR) can be interpreted as the difference in the number of cycles of ACT associated with survival. For example, in the non-adjusted model, 0.82 HR means that each additional cycle of chemotherapy was associated with an 18% decrease in the patients’ risk of death (0.82, 95% CI 0.72–0.93) while in the minimum-adjusted model (model 2), an ACT increase by 1 cycle resulted in a 16% decrease in the patients’ risk of death (0.84,95% CI 0.74–0.95). The risk of death was decreased by 26% (0.74, 95% CI 0.61–0.89) for each additional cycle of ACT in the fully adjusted model (model 3). We also converted the number of cycles of ACT from the continuous variable to the categorical variable for the sensitivity analysis. Patients who had more than 7 cycles of ACT had an 80% decrease in risk of death compared with patients who had no ACT (HR = 0.20, 95% CI 0.05–0.74).

**Table 2:** Univariate analyses of prognostic factors for 3-year cancer-specific survival

	Statistics	3-year CSS
	*N* (%)	HR (95%CI), *P* value
Gender		
Male	244 (61.00%)	Ref
Female	156 (39.00%),	1.27 (0.60, 2.72), 0.532
Age (year)	54.55 ± 12.81	1.01 (0.98, 1.04), 0.432
< 65	298 (74.50%)	Ref
≥ 65	102 (25.50%)	1.20 (0.53, 2.74), 0.6637
T stage		
T1–2	21 (5.25%)	Ref
T3–4	379 (94.75%)	1.35 (0.18, 9.97), 0.7669
N stage		
N0	182 (45.50%)	ref
N1	140 (35.00%)	2.66 (0.66, 10.62) 0.1670
N2	78 (19.50%)	15.55 (4.58, 52.82) < 0.0001
Differentiation		
Moderate to high	350 (87.50%)	Ref
Poor	50 (12.50%)	2.05 (0.83, 5.08), 0.1209
PLNR	0.145 ± 0.23	27.97 (9.88, 79.19), < 0.0001
< 0.145	286 (71.50%)	Ref
≥ 0.145	114 (28.50%)	7.56 (3.20, 17.88), < 0.0001
PNI	49.05 ± 5.51	0.93 (0.87, 1.00), 0.0403
PNI tertile		
T1	132 (33.00%)	Ref
T2	134 (33.50%)	0.34 (0.12, 0.95), 0.0398
T3	134 (33.50%)	0.54 (0.23, 1.30), 0.1686
NLR	3.21 ± 3.19	1.08 (1.00, 1.15), 0.0367
NLR tertile		
T1	133 (33.25%)	Ref
T2	133 (33.25%)	0.87 (0.29, 2.58), 0.7990
T3	134 (33.50%)	2.05 (0.83, 5.08), 0.1211
Number of ACT cycles	5.43 ± 3.17	0.82 (0.72, 0.93), 0.0022
0	36 (9.00%)	Ref
1–6	228 (57.00%)	0.28 (0.12, 0.67), 0.0044
7–12	136 (34.00%)	0.21 (0.07, 0.61), 0.0042
CCRT		
Without	147 (36.75%)	Ref
With	253 (63.25%)	1.09 (0.49, 2.43), 0.8296

*Abbreviations*: *PLNR* Positive lymph nodes ratio, *CA* Carcinoma antigen, *CEA* Carcinoembryonic antigen, *CCRT* Concurrent chemoradiotherapy, *ACT* Adjuvant chemotherapy, *PNI* Prognostic nutritional index, *NLR* Neutrophil-lymphocyte ratio

**Table 3:** Independent prognosis analysis of ACT cycles on 3-year CSS

	Non-adjusted	Adjust I	Adjust II
	HR (95% CI), *p*	HR (95% CI), *p*	HR (95% CI), *p*
No. of ACT cycles	0.82 (0.72, 0.93), 0.0022	0.84 (0.74, 0.95), 0.0061	0.74 (0.61, 0.89), 0.0016
0	Ref	Ref	Ref
1–6	0.28 (0.12, 0.67), 0.0044	0.43 (0.16, 1.15), 0.0929	0.33 (0.11, 0.94), 0.0380
7–12	0.21 (0.07, 0.61), 0.0042	0.24 (0.08, 0.75), 0.0138	0.20 (0.05, 0.74), 0.0160

Non-adjusted model adjusted for: none

Adjust I adjust for: gender; age; N stage; T stage

Adjust II adjust for: gender; age; N stage; T stage; differentiation; CEA; CA199; PNI; NLR; CCRT; PLN

*Abbreviations*: *PLNR* Positive lymph nodes ratio, *CA* Carcinoma antigen, *CEA* Carcinoembryonic antigen, *CCRT* Concurrent chemoradiotherapy, *ACT* Adjuvant chemotherapy, *PNI* Prognostic nutritional index, *NLR* Neutrophil-lymphocyte ratio

### The nomogram and its predictive performance

**Fig. 2:**
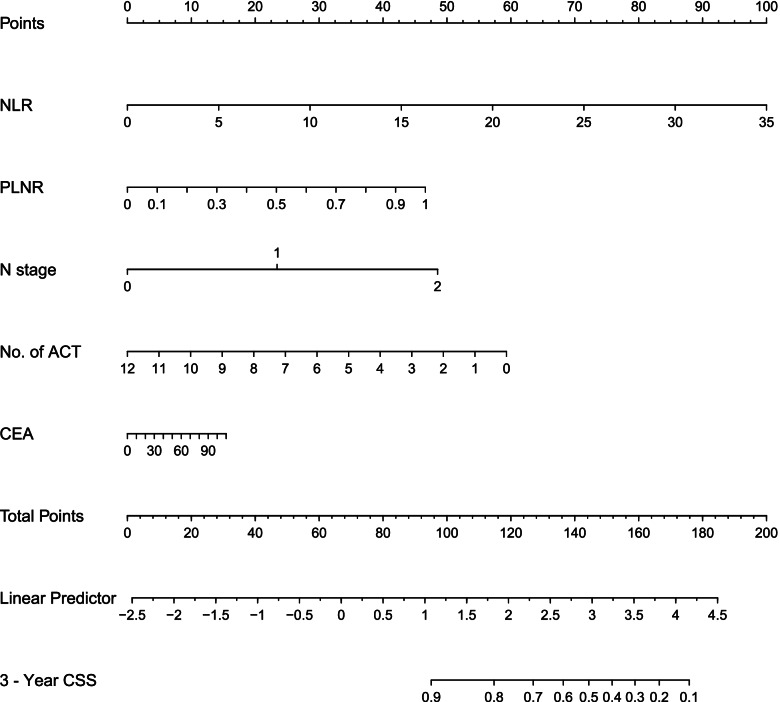
Nomogram predicting 3-year CSS in stage III and high-risk stage II rectal cancer patients. Abbreviations: CEA, carcinoembryonic antigen; PLNR, positive lymph nodes ratio; NLR, neutrophil-lymphocyte ratio; ACT, adjuvant chemotherapy

The study was adopted according to the Transparent Reporting of a Multivariable Prediction Model for Individual Prognosis or Diagnosis guidelines [17]. Based on the stepwise screening, factors related to the patients' 3-year CSS (N stage, PLNR, CEA, NLR, and the cycles of ACT) were included to establish a nomogram using the R software (Fig. 2).

**Fig. 3:**
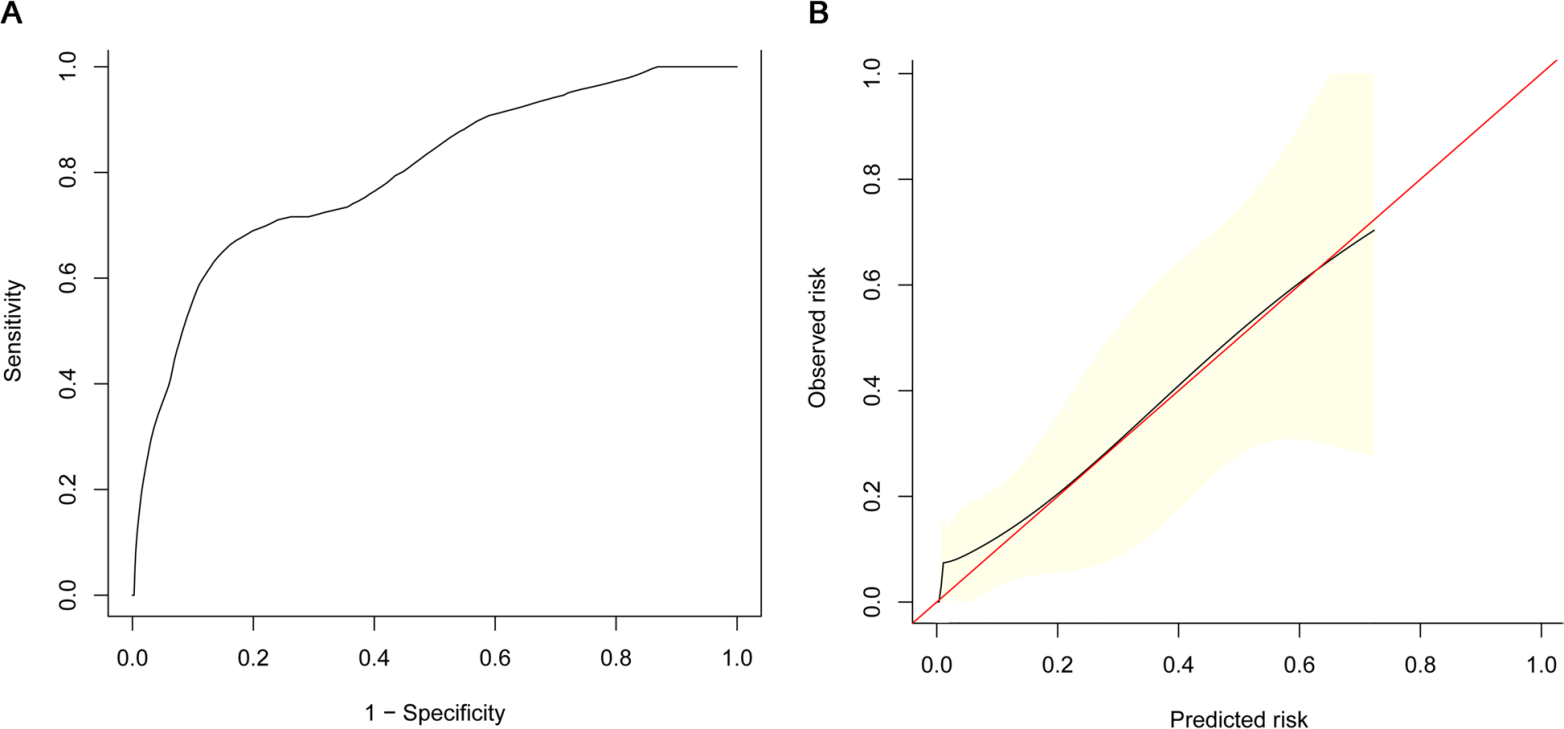
Internal validation of prognostic prediction for the nomogram. **A** Receiver operating characteristic; consistency index = 0.827. **B** Calibration plot. Black lines, nomogram-predicted probabilities; red line, the ideal line; yellow area, 95% confidence intervals

The nomogram has a consistency index of 0.827 [95% CI = 0.721–0.896]. The area under the curve of the model from the observed data (nomogram) was 0.803 (Fig. 3A). The time-dependent AUC was > 0.7 for the prediction of CSS, indicating favorable discrimination by the nomogram. The bootstrap resampling method was used as the internal validation and the calibration curve was drawn. The calibration curves of the nomogram showed high consistencies between the predicted and observed survival probability (Fig. 3B). In summary, the nomogram for rectal cancer patients had considerable discriminative and calibrating abilities.

### The establishment of the decision tree model by the recursive partitioning analysis

**Fig. 4:**
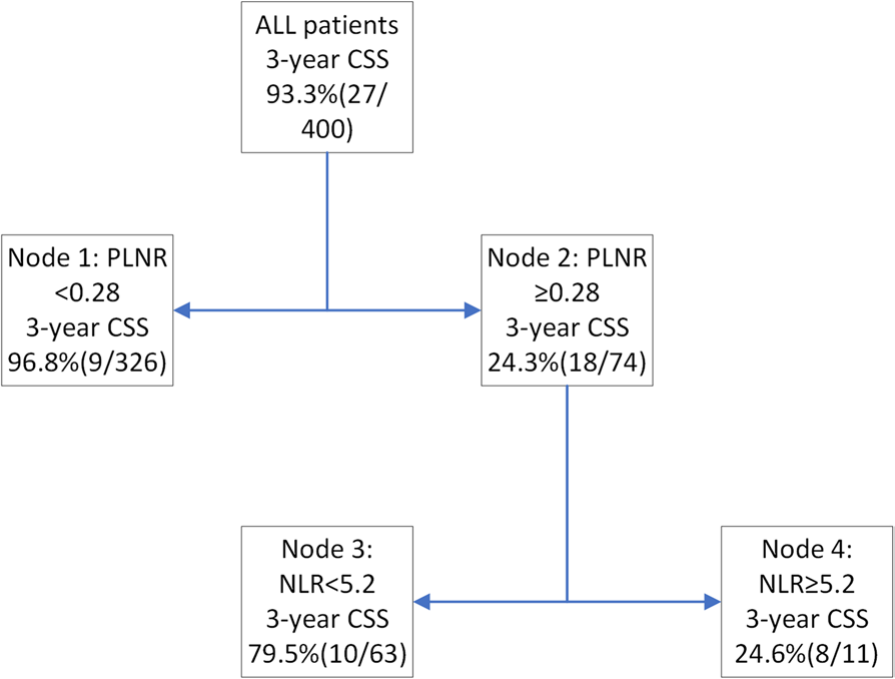
Decision tree model for prediction of 3-year CSS generated by the recursive partitioning analysis

RPA identified two predictors (PLNR, NLR) that stratified patients based on 3-year CSS (Fig. 4). Node 1 (PLNR < 0.28) was used to identify the low-risk group and the intermediate-risk group included node 2 and node 3 (PLNR ≥ 0.28 and NLR < 5.2). Nine of the 326 patients (2.76%) from the low-risk group and 10 of the 63 (15.9%) patients from the intermediate-risk group eventually died. Node 2 (PLNR ≥ 0.28) and Node 4 (NLR ≥ 5.2) were included to identify the high-risk group. Overall, 72.7% (8/11) of the patients from the high-risk group died in the first 3 years of treatment.

**Fig. 5:**
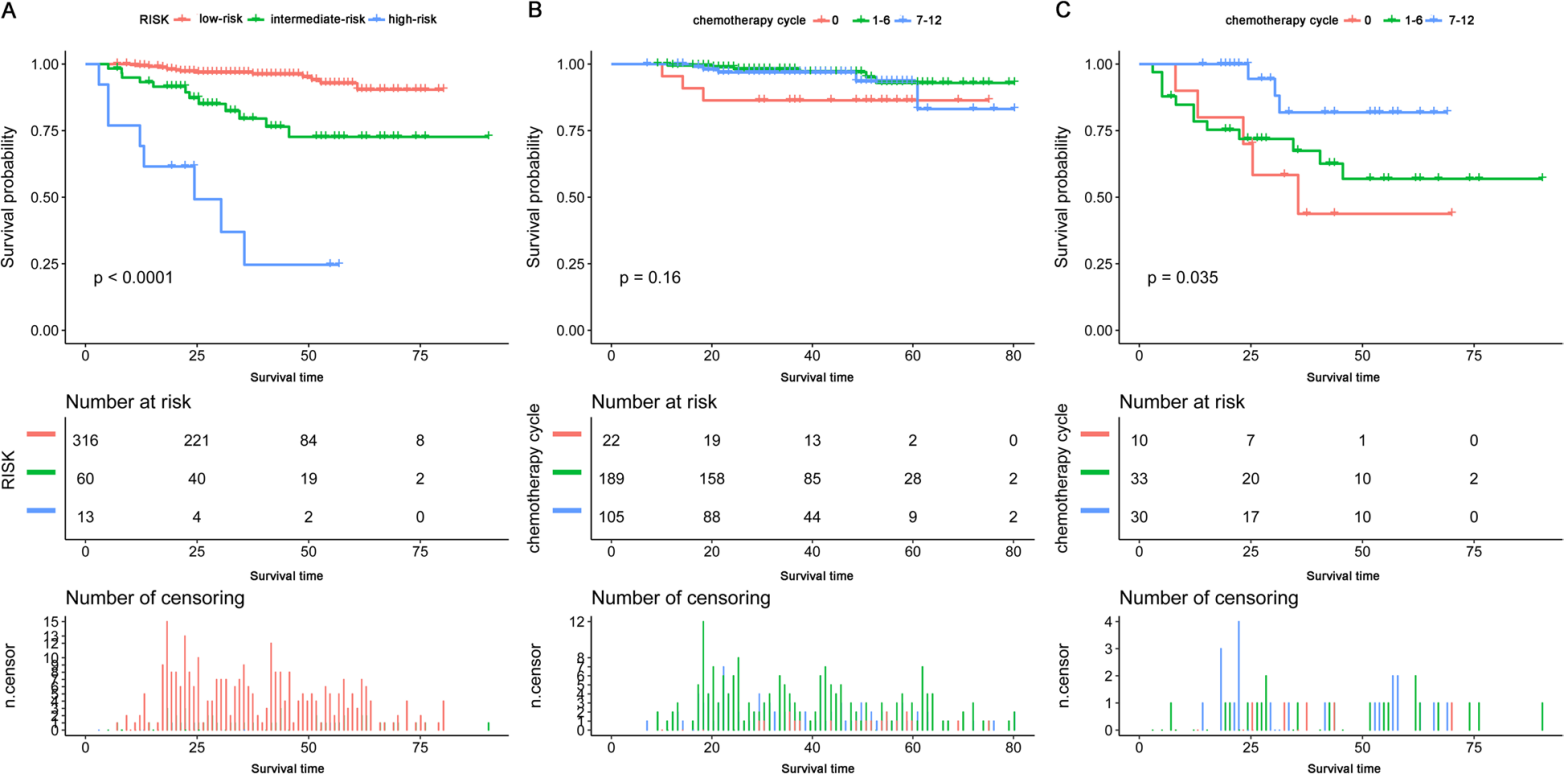
Kaplan-Meier curves (**A**) for risk groups stratified by the recursive partitioning analysis; (**B**) in patients who received various numbers of adjuvant chemotherapy cycle with PLNR < 0.28; (**C**) in patients who received various numbers of adjuvant chemotherapy cycle with PLNR ≥ 0.28

Figure 5 A illustrates a significant difference in survival between the low-risk group, the intermediate-risk group, and the high-risk group (3-year CSS: 0.968 vs. 0.795 vs. 0.246, *P* < 0.0001). Due to the limitation in the number of patients in the high-risk group, we combined the high-risk and intermediate-risk groups for the subgroup analysis. The subgroup analysis, based on the number of cycles of ACT, revealed no significant benefit from ACT of 7 cycles or more in the low-risk group (*P* = 0.16) (Fig. 5B). However, in the high-risk and intermediate-risk groups, the patients who received no less than 7 cycles of ACT had a better prognosis than those who received no chemotherapy or fewer than 7 cycles (*P* = 0.035) (Fig. 5C).

## Discussion

In this study, we found that with multiple regression analyses adjusted for confounding factors, the number of cycles of ACT was an independent prognostic factor affecting 3-year CSS in stage III and high-risk stage II rectal cancer in patients who had received TME. Our multiple regression model adjusted for a series of confounding factors, including the patients’ demographic characteristics, tumor characteristics, and clinical interventions (radiotherapy) to better assess the independent effect of the number of cycles of ACT on prognosis. Chang et al. [18] found that Chinese rectal cancer patients benefited from more than 8 cycles of capecitabine and oxaliplatin, which is consistent with our results in high-risk and intermediate-risk groups. Although we recommended CCRT, especially NACR, to all locally advanced rectal cancer patients in the original treatment plan based on internationally recognized treatment guidelines, 147 (147/400) patients refused to receive CCRT either before or after surgery according to the clinical records of enrolled patients. Of these 147 patients, 118 cases were T1-2/N1 or T3N0, 17 cases were T2N2, and 12 were T3N1. They all had received ACT after TME surgery. Previous studies suggested that in addition to ACT, combined CCRT does not prolong disease-free survival or overall survival in rectal cancer patients with a low risk of local recurrence (T1–2/N1 or T3N0) [19, 20]. Therefore, patients free of high-risk local recurrence factors (T3N2 or T4), even without CCRT, were enrolled in our analysis.

We found that the risk of death was reduced by 26% for each additional cycle of chemotherapy (HR = 0.76, 95% CI: 0.61–0.89, *P* = 0.0016). Although our participants received the oxaliplatin/fluorouracil regimen (FOLFOX), which was different from the regimen that Chang et al. [18] used, we found that increasing the chemotherapy cycle was beneficial for prognosis in our patients. In our hospital, most patients received the FOLFOX regimen. To reduce the interaction effect of chemotherapy regimens, as has been previously reported [21], we excluded 26 patients who received chemotherapy with capecitabine and oxaliplatin (XELOX) or fluorouracil/capecitabine. Although 91% of the patients received adjuvant postoperative chemotherapy and the original treatment plan was 6 months in duration, only 34% of these patients completed more than 6 cycles, which is possibly due to the intolerance of the medications. Thus, we will consider giving systemic chemotherapy treatment to our patients before surgery in future research, since the concept of total neoadjuvant chemotherapy is well established and known to show that in the total neoadjuvant approach, more patients were able to finish their treatments.

We also developed a nomogram appropriate for stage III and high-risk stage II rectal cancer in patients. Previous prognostic models of colorectal cancer included CEA, NLR, N stage, PLNR, and other indicators [21–23] but no studies have included the number of cycles of ACT as a variable in the prognosis model. Based on our Cox multiple regression model adjusted for the confounding factors, the number of cycles of ACT is an independent prognostic factor. After a stepwise method of variable screening, the number of cycles of ACT was also found to be suitable for inclusion in the nomogram model. In addition to the patients’ baseline characteristics, the inclusion of subsequent clinical interventions in establishing a prognostic model may help in comprehensively assessing the prognosis of the disease.

The adverse effect of ACT on quality of life is another concern in these patients. In a previous trial reported by Schmoll et al. [24], among postoperative colon cancer patients who received 8 cycles or more of capecitabine and oxaliplatin, grade 3/4 acute toxicity occurred in 56.5% of the patients. Therefore, it is necessary to develop an individualized treatment for patients undergoing postoperative chemotherapy. Another recent study [21] also reported that 3-month adjuvant chemotherapy was not inferior to 6 months of treatment for stage III colon cancer patients who received a capecitabine and oxaliplatin regimen. However, the authors conducting this study found no evidence that 3-month chemotherapy could replace 6-month chemotherapy in the FOLFOX regimen. In our study, for stage III and high-risk stage II population that was given the FOLFOX regimen, two factors (PLNR and NLR) were identified by RPA and a subgroup analysis referring to risk stratification was performed. We demonstrated that patients in the high-risk and intermediate-risk groups (PLNR ≥ 0.28) that received chemotherapy of at least 7 cycles had a significant improvement in survival. Previous studies have reported that with the increase in PLNR, the survival rate of colorectal cancer patients continues to decline due to the increased probability of distant metastases [25–28]. This finding is consistent with our conclusion that patients with high PLNR may require longer adjuvant chemotherapy. Our results also revealed that patients with low and intermediate risk (PLNR < 0.28) may not be eligible for longer durations of chemotherapy (7 or more cycles). This observation may also suggest that patients with a low probability of lymph node metastasis have a lower risk of distant metastases after local radical surgery, such as TME.

The present study made three original contributions. Firstly, we demonstrated the independent effect of the number of cycles of ACT on the prognosis of stage III and high-risk stage II rectal cancer. Secondly, we established a nomogram to predict the 3-year CSS of such patients. Thirdly, we used RPA to identify rectal cancer patients who might benefit from a longer duration of chemotherapy. The similar use of RPA, which has not yet been published, might help in treatment decision-making in patients with rectal cancer.

We acknowledge that our study has limitations. Firstly, it is a single-center study in China, with inevitable bias in the case selection; validation in other centers or other populations worldwide is needed. Secondly, due to the limitation in the number of patients in the high-risk group (11 patients), most patients (63 patients) were from the intermediate-risk group, we need to expand the sample size to verify our conclusions. Finally, for the patients who have not completed 6 months of chemotherapy, the data on the reasons for stopping chemotherapy are incomplete.

## Conclusions

In conclusion, for stage III and high-risk stage II rectal cancer patients, the number of ACT cycles after TME is an independent prognostic factor when adjusted for the confounding factors. We developed a novel and practical nomogram incorporating the N stage, CEA, NLR, PLNR, and the cycles of ACT that accurately predicts patients’ 3-year CSS. For patients with PLNR ≥ 0.28, no less than 7 cycles of ACT are needed to significantly reduce the risk of death.

## Data Availability

All datasets generated and/or analyzed during this study are included in this article.
